# Chemical and Biological Properties of C-Point Obturation Cones

**DOI:** 10.3390/biomimetics10060409

**Published:** 2025-06-18

**Authors:** Marina Angélica Marciano, Paulo Jorge Palma, Ana Cristina Padilha Janini, Brenda Fornazaro Moraes, Thiago Bessa Marconato Antunes, Ribamar Lazanha Lucateli, Bruno Martini Guimarães, Mariza Akemi Matsumoto, Diana Bela Sequeira, Talita Tartari, Brenda Paula Figueiredo Almeida Gomes, Marco Antonio Hungaro Duarte

**Affiliations:** 1 Department of Restorative Dentistry-Endondontic, School of Dentistry of Piracicaba, State University of Campinas, São Paulo 13414-903, Brazil; a235459@dac.unicamp.br (A.C.P.J.); b272366@dac.unicamp.br (B.F.M.); t157403@dac.unicamp.br (T.B.M.A.); talitat@unicamp.br (T.T.); bpgomes@unicam.p.br (B.P.F.A.G.); 2 Institute of Endodontics, Faculty of Medicine, University of Coimbra, Coimbra, Portugal and Center for Innovation and Research in Oral Sciences (CIROS), 3000-075 Coimbra, Portugal; ppalma@uc.pt; 3 Department of Surgery, Dental School of Ribeirão Preto, University of São Paulo—USP, Ribeirão Preto, São Paulo 14040-900, Brazil; ribamarlazanha@gmail.com; 4 School of Dentistry, Federal University of Alfenas, Alfenas 37130-001, Brazil; bruno.guimaraes@unifal-mg.edu.br; 5 Department of Basic Sciences, Dental School of Araçatuba, State University of São Paulo, São Paulo 16015-050, Brazil; mariza.matsumoto@unesp.br; 6 CNC-Center for Neuroscience and Cell Biology, University of Coimbra, 3004-504 Coimbra, Portugal; dianasequeira@fmed.uc.pt; 7 Department of Dentistry, Endodontics and Dental Materials, Dental School of Bauru, University of São Paulo—USP, São Paulo 17012-901, Brazil; mhungaro@fob.usp.br

**Keywords:** biocompatibility, biomaterials, materials science, root canal filling

## Abstract

This study evaluated the chemical composition and subcutaneous tissue biocompatibility of C-Point, a root canal filling material, compared to ProTaper gutta-percha cones (control). Material characterization was conducted using scanning electron microscopy with energy-dispersive spectroscopy (SEM-EDS). For biocompatibility assessment, both materials were implanted subcutaneously in the dorsal connective tissue of sixteen albino rats (n = 8 per group). Histological evaluation of inflammatory infiltrate intensity was performed at 30 and 60 days post-implantation, with statistical analysis (significance set at *p* < 0.05). SEM-EDS analysis revealed distinct elemental compositions: C-Point primarily contained zirconium and cobalt ions, while gutta-percha cones demonstrated a strong zinc signature with trace amounts of barium, aluminum, and sulfur. Both materials exhibited similar particulate morphology with radiopaque inclusions. Histologically, no significant difference in inflammatory response was observed between C-Point and gutta-percha at any time point (*p* > 0.05). All specimens developed a fibrous encapsulation. The inflammatory profile showed temporal dynamics, with lymphocyte predominance during early stages that progressively diminished by the study endpoint. These findings demonstrate that while C-Point possesses a unique elemental profile dominated by zirconium, its tissue biocompatibility parallels that of conventional gutta-percha obturation materials. However, due to the absence of mechanical testing and the limited in vivo follow-up period, the long-term stability of the material remains uncertain.

## 1. Introduction

The success of endodontic therapy goes beyond the removal of infected tissue; it also depends on the choice of materials capable of effectively sealing the root canal system and promoting the healing of periapical tissues [1]. To this end, the filling combines solid components with fluid materials, called sealing cements. Since these materials remain in direct contact with the tissues for long periods, it is essential that they present low toxicity and contribute to biological repair [2,3]. In addition, hermetic sealing is essential to prevent reinfection and ensure the maintenance of the dental element and periapical tissues over time [2,4].

However, toxic byproducts and chronic inflammatory reactions associated with certain filling materials can compromise periapical healing and generate long-term clinical failures [5,6]. Therefore, it is essential to understand the biological behavior and physicochemical properties of these materials, especially considering that, in some unintentional cases, they can go beyond the root apex and come into direct contact with adjacent connective tissues [7].

Among solid materials, gutta-percha has established itself as the standard over the last few decades [8]. Its wide clinical adoption is due to its biocompatibility, ease of handling and good sealing capacity associated with appropriate root canal sealers [7,9,10]. Although it adapts reasonably well to the canal walls, small spaces are still present, so it must be associated with the use of sealers to ensure complete sealing, considering further that studies demonstrate that the surface characteristics of these materials and the degree of contact with the dentin walls influence the inflammatory responses of the tissues [11].

Clinical studies indicate that failure to seal the root canal system is still one of the main causes of failure in endodontic treatments, even with adequate instrumentation and irrigation techniques [12]. In the search for effective solutions and improvements, several alternatives have been studied in relation to root canal filling. Gutta-percha coated with silver nanoparticles, for example, presents additional antibacterial properties and sealing performance similar to that of conventional gutta-percha [13]. The Resilon/Epiphany system, a thermoplastic polymer proposed for adhesion to dentin, is justified by its potential ability to reinforce the tooth structure [14]—although it has demonstrated lower long-term stability and greater degradation when compared to traditional gutta-percha [15,16]. To date, the available evidence does not support the replacement of the traditional combination of gutta-percha and cement [17].

Recently, the C-Point obturation system (marketed as ProPoint in the SmartSeal system, DRFP Ltd., United Kingdom) has emerged as a hydrophilic alternative. C-Point cones have a nylon polymer core and an expandable outer layer composed of acrylonitrile and vinylpyrrolidone copolymers [18]. Upon contact with the moisture in the canal, these materials expand laterally, favoring the adaptation of the sealer to the canal irregularities, allowing a complete seal. This expansion mechanism is controlled and restricted for the laterals, while axially, the dimension is maintained, avoiding the risk of apical extrusion [9,19]. Furthermore, mechanical studies on this material have shown that it has the ability to increase the resistance of the dentin wall to fracture, when compared to gutta-percha [20].

As with any endodontic material, biocompatibility is an extremely important aspect. Biocompatibility is an essential characteristic for any material used in endodontic procedures. It is the ability of the material to interact with living tissues without causing adverse effects, such as necrosis, persistent inflammation, or toxicity. To evaluate this property, animal models—especially the Wistar rat—are widely used in preclinical research. The Wistar rat is one of the most widely used species in biomedical research for several reasons, such as ease of handling, well-characterized immunological responses, reproducibility and low rate of biological variation, and ethical acceptance. Although gutta-percha presents good tissue tolerance even after decades of use, some alternative materials proposed by manufacturers have demonstrated significant inflammatory potential in studies with connective tissues [21], which reinforces the importance of continuous investigations at the same pace at which new materials are launched in the dental market.

In parallel with the development of new cones, the endodontic industry has also made progress in the formulation of sealing cements. Calcium silicate-based materials have stood out for their high biocompatibility, antibacterial potential, release of calcium and hydroxyl ions, and ability to induce the formation of hard tissues [21]. Another novelty in relation to endodontic cements was the incorporation of bioactive glass. These materials release ions such as calcium, phosphorus, and silicon, which promote tissue regeneration and combat bacterial proliferation [22]. Thus, they not only physically seal the canal, but also stimulate biological repair—a concept aligned with the current trend of developing materials with sealing and biological functions.

Several methods can be used to evaluate the properties of endodontic materials. Regarding chemical and structural properties, scanning electron microscopy (SEM) and energy-dispersive X-ray spectroscopy (EDS) are of paramount importance. SEM allows the observation of surfaces and interfaces in high resolution, revealing in rich detail the morphology of the structures involved. Energy Dispersive X-ray Spectroscopy (EDS or EDX) is an analytical technique coupled with scanning electron microscopy (SEM), which allows the identification and quantification of chemical elements present on the surface of a material. Despite all these advances, there is still no ideal obturation material. The desired material must combine biocompatibility, antimicrobial properties, dimensional stability, ease of application, and the ability to promote a long-lasting seal [23].

In view of this, laboratory and clinical studies, especially with long-term monitoring, are essential to evaluate the development of innovative systems such as C-Point and bioactive cements in comparison with materials traditionally used in endodontic clinics. Studies addressing C-Point’s biological and physical-chemical properties are still scarce, especially in direct comparison with conventional materials such as gutta-percha. This study aims to investigate the biological response and chemical properties of C-Point obturators in relation to conventional endodontic obturation materials.

Beyond addressing these specific outcomes, the present work also fits within a broader context of innovation in endodontic materials, particularly through a biomimetic perspective. This study enriches the growing field of research dedicated to the development of biomimetic materials in dentistry, investigating the chemical composition and biological performance of C-Point obturation cones in relation to traditional gutta-percha. Biomimetics, an interdisciplinary field, seeks to create technologies that combine mechanical function with structural efficiency and biological compatibility, inspired by what occurs in nature [24]. In this context, understanding how endodontic materials interact with living tissues is crucial to develop solutions that go beyond simple sealing of the root canal system and actively support biological functions, such as tissue repair, in the short and long term.

The hydrophilic expansion mechanism of C-Point, designed to improve adaptation to irregularities and restore a canal-filling structure through lateral expansion, exemplifies a biomimetic approach, dynamically reacting to the moisture naturally present in the root canal environment. However, this functional behavior must be analyzed in conjunction with the chemical and mechanical stability and the long-term biological response of the material. As will be explored in more detail in this study, initial observations suggest that, despite its different formulation, with the inclusion of zirconium and cobalt, C-Point can promote a tissue response comparable to that of gutta-percha, including signs of fibrous encapsulation and low levels of inflammation.

These considerations reflect key principles of biomimetics, which emphasize the integration of synthetic materials into biological environments in a functional, harmonious manner that supports biological processes. By combining structural characterization with in vivo biological evaluation, this study aims to expand the understanding of how new endodontic materials interact with host tissues, reinforcing their potential as bioinspired alternatives to established options in clinical practice.

Thus, this work demonstrates how endodontic technologies are evolving into materials that go beyond occupying space; they interact biologically, adapt to physiological conditions, and seek long-term compatibility and mimicry of natural structures. In doing so, they align with the broader vision of biomimicry: not just restoring function, but achieving integration with living systems.

## 2. Materials and Methods

Two different obturation systems were selected: ProTaper gutta-percha and C-Point cone (EndoTechnologies, Shrewsbury, MA, USA).

### 2.1. Microscopy and Elemental Analysis

The samples were sputter-coated with a thin layer of carbon to enhance conductivity prior to examination using a JEOL JSM 5600 Lv scanning electron microscope (Tokyo, Japan). Imaging was performed at varying magnification levels utilizing both secondary electron and backscattered electron detection modes. Additionally, compositional analysis was conducted through energy-dispersive X-ray spectroscopy (EDS) to identify and characterize elemental constituents within the specimens.

### 2.2. Sample Size

Statistical power analysis was conducted using G*Power 3.1 for Mac (Heinrich Heine University, Düsseldorf) to compare two independent means. The parameters included an estimated standard deviation of 2.7, a detectable minimum difference of 8.7, a power (1-β) of 0.80, and a significance level (α) of 0.05.

The number of animals was defined in accordance with the 3Rs guidelines (Replacement, Reduction, Refinement), minimizing the excessive use of animal models. All animals included presented good systemic conditions, with no signs of infection or weight change during the acclimatization period.

Sixteen healthy adults male Wistar rats (*Rattus norvegicus*), aged 12 weeks and weighing approximately 300 g, were included in the study, with 8 animals per tested material (four animals per experimental period).

### 2.3. Biocompatibility

Ethical approval was obtained (CEP 014-2014), and the research adhered to the ARRIVE (Animal Research: Reporting In Vivo Experiments) Guidelines. The animals were acclimatized under controlled conditions in a vivarium. They were housed in sanitized, shared plastic cages (two animals per cage) with a maintained temperature of 20–22 °C. Standard commercial rodent chow and water were provided ad libitum throughout the acclimatization period. A 12 h light/dark cycle was maintained for circadian regulation.

The choice of implantation in the dorsal subcutaneous region was based on ease of surgical access, reduced risk of infection and low mobility, allowing ideal conditions for histological analysis. The procedures were conducted by a team trained in experimental microsurgery.

Prior to surgery, the animals were anesthetized via intraperitoneal injection using a mixture of ketamine hydrochloride (anesthetic) and xylazine hydrochloride (sedative and muscle relaxant). After confirming complete anesthesia, the dorsal area of each rat was shaved and disinfected with povidone-iodine (PVPI). Two longitudinal incisions (~1 cm each) were then made along the midline of the back using a sterile #15 scalpel blade (Swan-Morton, Sheffield, UK). Using blunt dissection scissors, the subcutaneous tissue was carefully separated to create two small pockets (one on each side) for implant placement (2 implants per animal). Two sterile polyethylene tubes measuring 10 mm in length, 1 mm in internal diameter and 1.6 mm in external diameter, filled with the same material implanted in the alveolar region, were implanted in the dorsal subcutaneous region while still under anesthesia.

The rats were randomly allocated into four experimental groups (n = 4 per group) based on the material being tested.

Postoperative analgesia was ensured by intraperitoneal administration of dipyrone sodium (500 mg/mL) at a dose of 100 mg/kg body weight. To identify the animals, markings were performed by piercing the ears using an Ainsworth perforator (Duflex, SS White, Rio de Janeiro, Brazil).

At the conclusion of the 30- and 60-day experimental periods, euthanasia was humanely performed through intraperitoneal administration of an overdose of ketamine hydrochloride (240 mg/kg) and xylazine hydrochloride (30 mg/kg). Euthanasia was performed in a private room with low lighting, clean, noise-free, and away from other animals to avoid stress. One animal was euthanized at a time, with the corpse removed from the room and the room, as well as the objects used, cleaned before the next animal entered. The animal’s death was confirmed by observing the following signs: absence of respiratory movement (apnea); absence of heartbeat (asystole) using a stethoscope; absence of pulse; pale mucous membranes; and loss of corneal reflex.

Following extraction, the peri-implant subcutaneous tissue specimens were immediately fixed in neutral-buffered 10% formalin solution (Sigma-Aldrich, Saint Louis, MO, USA, pH 7.0) for a minimum of 48 h. After fixation, tissues underwent thorough rinsing under continuous water flow for 24 h, followed by progressive dehydration using an ascending ethanol series.

The dehydrated samples were individually embedded in paraffin blocks and sectioned at 5 μm thickness using a microtome. These sections were mounted on glass slides and stained using standard hematoxylin and eosin (H&E) protocols.

All histological assessments were performed by a board-certified pathologist using an Olympus light microscope (São Paulo, Brazil) at 40× magnification. For quantitative analysis of inflammatory infiltrate, thirty representative microscopic fields per sample were systematically evaluated. To validate findings, all measurements were performed in duplicate to confirm methodological reproducibility.

### 2.4. Statistical Analysis

Data were assessed for normal distribution using the D’Agostino–Pearson test. Since the data met the parametric criteria, one-way ANOVA with Tukey’s post-test was used. Values of *p* < 0.05 were considered statistically significant.

## 3. Results

### 3.1. Characterization

**Figure 1:**
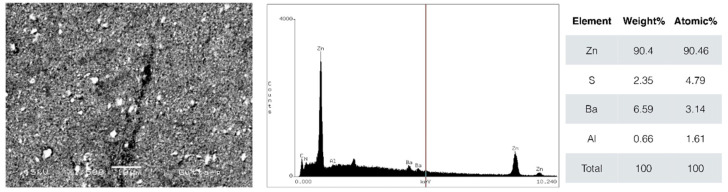
Scanning electron microscope (SEM) micrographs in backscatter mode of gutta-percha cone. Energy-dispersive X-ray spectroscopy (EDS) shows elemental composition. Magnification 1500.

**Figure 2:**
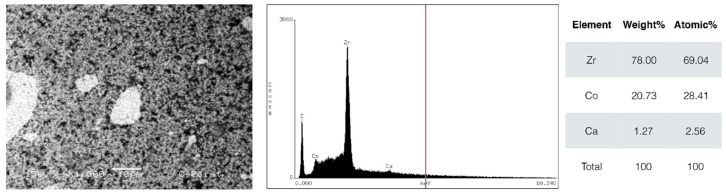
Scanning electron microscope (SEM) micrographs in backscatter mode of C-point cone. Energy-dispersive X-ray spectroscopy (EDS) shows elemental composition. Magnification 1500.

Scanning electron micrographs of the gutta-percha cone are shown in Figure 1, and those of the C-Point in Figure 2. Gutta-percha cone exhibited higher intensity of zinc ions peak (Zn—90%) and with traces of barium (Ba), aluminum (Al), and sulfur (S). The C-Point exhibited predominance of zirconium ions (Zr—78%), followed by cobalt (Co—20%). The particles of both obturating materials were similar in SEM analysis, with the presence of radiopaque structures inside the bulk.

**Figure 3:**
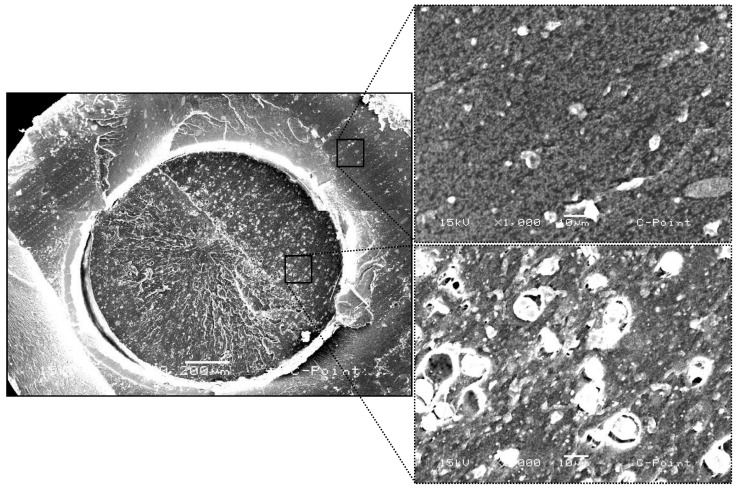
Scanning electron microscopy (SEM) images of the C-Point endodontic obturation cone, showing its layered structure. The low-magnification image (70) reveals the distinct outer layer and inner core, suggesting differences in composition or porosity. Higher magnification (1000) highlights the dense and homogeneous texture of the outer layer, contrasted with the more porous inner region.

The scanning electron microscopy (SEM) images illustrate the structural characteristics of C-Point (Figure 3). In the low-magnification micrograph (70), a transverse section of the cone is displayed, revealing its layered architecture. The outer layer appears distinct from the inner core, suggesting potential differences in composition or porosity. At higher magnification (1000), detailed images of both the outer and inner layers are provided. The outer layer exhibits a relatively dense and homogeneous texture, whereas the inner region presents a more porous structure.

### 3.2. Tissue Response

**Figure 4:**
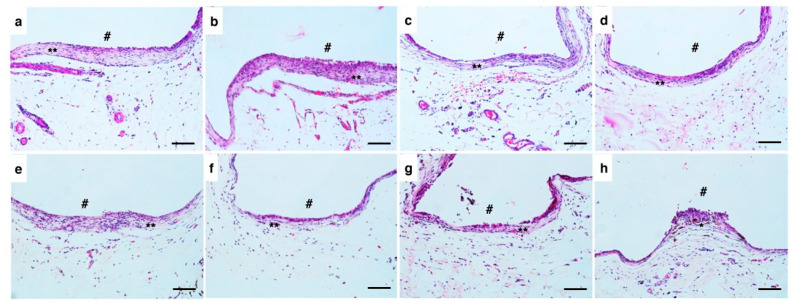
Microscopic representative specimens at 30 days of analysis are showed in the figures (**a**–**d**). ProTaper gutta-percha point (**a**,**b**), C-Point cone (**c**,**d**). Close to the material (#)*,* areas with fibrous connective tissue in a capsule aspect were visualized (**), with a slight infiltrate of inflammatory cells. Microscopic representative specimens at 60 days of analysis are shown in the figures (**e**–**h**). ProTaper gutta-percha point (**e**,**f**) and C-Point cone (**g**,**h**). In general, there was formation of a fibrous capsule (**) close to the material (#) (40 magnification).

As detailed in Table 1, the inflammatory infiltrate was characterized by median values with corresponding minimum and maximum ranges. Figure 4 illustrates representative histological sections from each experimental group at different time points.

Microscopic examination demonstrated consistent connective tissue neoformation surrounding all implanted materials. The inflammatory profile showed temporal dynamics, with lymphocyte predominance during early stages, which progressively diminished toward the study endpoint. At the final evaluation period, specimens exhibited mature capsular tissue organization.

Statistical comparison of inflammatory parameters (median and range values) revealed no significant differences (*p* > 0.05) between material groups at any time point, as determined by our quantitative analysis.

## 4. Discussion

The selection of materials used in root canal obturation is an essential factor in determining the outcomes of endodontic treatment. The fundamental functions of obturation require materials capable of preventing reinfection of the root canal system and promoting the healing of periapical tissues [25]. Gutta-percha is traditionally the main material used in root canal obturation due to its established sealing properties and its radiographic visibility, which facilitates image interpretation. Newer materials, such as C-Point, emerge as promising alternatives due to their differentiated physical and compositional characteristics.

The samples were chemically evaluated using the SEM/EDS technique to demonstrate the importance of the chemical characterization of the materials, compared to the biological analysis performed. Scanning electron microscopy (SEM) analyses revealed significant structural differences between C-Point and conventional gutta-percha. In cross-section, C-Point presents a layered structure with a denser external surface and a less compact internal region composed of materials of different natures. This arrangement and the special composition of C-Point allow it to expand laterally when exposed to moisture, promoting better sealing of the root canal, as indicated in studies [26]. This technology is justified considering that endodontic success depends on adequate sealing, blocking bacterial infiltration and reducing the risk of periapical inflammation [23,27].

According to energy dispersive spectroscopy (EDS) analysis, the materials tested presented distinct chemical compositions. C-Point presented a high zirconium (Zr) content, approximately 78%, which improves radiographic visibility and possibly provides good mechanical resistance. The main elements detected in gutta-percha were zinc (Zn, 90.4%), sulfur (S, 2.35%), barium (Ba, 6.59%), and aluminum (Al, 0.66%). Barium, added to gutta-percha, contributes to greater radiopacity [28].

There are reasons to pay special attention to the presence of zirconium and cobalt in the composition of C-Point cones, due to potential long-term biological effects. Although zirconium is generally considered inert, research has shown that zirconium-one particles can induce inflammatory responses, including increased expression of proinflammatory cytokines such as IL-1β and TNF-α [29]. Therefore, research is needed to explore the long-term effects of ions released by C-Point and its contact with periapical tissue.

To achieve clinical success, the materials used in obturation must be biocompatible, since they come into direct contact with the periapical tissues. Laboratory procedures allow the evaluation of the biological response to different materials, and recent studies have compared gutta-percha compositions with alternative solutions [8]. In vitro research demonstrated that both conventional gutta-percha and C-Point cones were toxic to periodontal ligament fibroblasts, while bioceramic-based points did not exhibit cytotoxic effects [7].

The combination of nylon polymer core and acrylonitrile and vinylpyrrolidone co-polymer outer layer in C-Point raises numerous questions about how compatible and stable this material remains in the body. The cytotoxic effects of C-Point eluates toward MDPC-23 cells derived from rat dental papilla were tested in previous research [9]. During the initial seven days of testing this hydrophilic material demonstrated more toxic properties compared to typical gutta-percha. The cytotoxicity levels progressively decreased to match those of gutta-percha after an initial duration of testing. Quantitative real-time PCR assessment demonstrated that the cellular mineralization potential was maintained throughout the in vitro experimental period.

Histological evaluation of the specimens after 30 and 60 days showed areas with fibrous connective tissue and mild inflammatory infiltrate, with no statistically significant differences between the groups, even with a tendency towards slightly higher median values in the C-Point group. The implantation of biomaterials generates specific reactions in host tissues, starting with tissue injury, blood–material contact, and formation of a provisional matrix, progressing to acute and chronic inflammation, formation of granulation tissue, foreign body reaction and, finally, fibrous encapsulation [30]. Biocompatible materials often induce the formation of fibrous capsules, since, even though they are biocompatible, the organism tends to isolate foreign substances [30]. The tissue reactions observed were similar between C-Point and the gutta-percha control, indicating that C-Point does not trigger significant adverse responses beyond those expected during natural healing, considering that the presence of a mature fibrous capsule after 60 days indicates a favorable biological adaptation to the material.

Performing biocompatibility tests on animals, especially Wistar rats, is a fundamental step in the research of new endodontic materials, such as cements, sealers, and intracanal medicaments. These tests allow the evaluation of the initial and late biological response of living tissues to contact with the material before its use in humans. The choice of the animal model (Wistar rats) was justified by the ability to analyze the contact between the material and tissue over time, providing a representation of human conditions (approximately 3 years for a 30-day implantation period). The selection of only male rats aimed to avoid potential hormonal bias. In this study, 12-week-old rats were chosen for surgery to correspond to a young age in humans. Another important factor is the weight uniformity required for the inclusion of animals in the study. For this reason, the weighing with normal values presented by all test and control animals included in the analysis represented a population with significantly similar weight. In addition, weight gain was constant as a consequence of the normal growth and development of the animals during the period analyzed.

The absence of significant differences between the groups demonstrates that, despite compositional and structural differences, C-Point presents a stimulus of biological behavior comparable to that of gutta-percha in subcutaneous experimental conditions.

The biological responses between C-Point and gutta-percha observed in in vitro and in vivo studies show similar results [7,9,18], but confirmation in long-term clinical studies remains essential. Large-scale randomized clinical trials should be conducted to validate the therapeutic outcomes and possible complications associated with the use of C-Point in different clinical settings.

Initial research shows promising results regarding the performance of C-Point, but aspects of its long-term behavior are not yet fully understood. Additional studies should investigate the clinical behavior of the material in different therapeutic situations. Despite previous studies indicating good results regarding C-Point, it is essential to perform prolonged evaluations to verify the stability of the polymeric material and the effects on its mechanical properties over time. Long-term evaluations are necessary to determine whether its polymeric composition degrades, affecting sealing and mechanical integrity. In addition, it is important to study the impact of the periapical environment on C-Point degradation and possible changes in its physicochemical characteristics with prolonged clinical use.

The current endodontic practice employs obturation materials in combination with sealers. According to the manufacturer’s recommendations, recent studies have shown that the use of C-Point with EndoSequence^®^ BC Sealer™ provides significantly higher bond strength compared to the traditional gutta-percha/AH Plus combination [18]. Despite this, the gutta-percha/AH Plus combination continues to be considered the gold standard for root canal obturation according to the literature [10]. Although the use of different materials is common in endodontic practice, the interaction of C-Point with various sealers still requires investigation, especially regarding chemical compatibility and bond strength.

Further studies should also investigate whether C-Point has antibacterial properties that may provide superior clinical effects. Evaluations of its resistance to biofilm formation and bacterial colonization may provide important data on its advantages over standard endodontic materials, considering the impact of bacterial persistence on treatment outcomes.

Although the animal model, Wistar rats, is widely accepted and ethically viable for evaluating the biocompatibility of endodontic materials, there are inherent limitations in extrapolating the results to clinical practice [31,32]. The subcutaneous environment, although complex, differs significantly from periapical tissues in terms of vascularization, cellular composition and, mainly, mechanical stress. In addition, immunological responses and metabolic rates differ, which may not fully reflect the biological behavior of the materials in the human periapical region [33]. Thus, although the findings indicate a favorable tissue response to C-Point, clinical studies are essential to validate its biocompatibility and performance under real conditions of endodontic use.

Future research on the biological response to C-Point should investigate the cytotoxic effects, material degradation mechanisms and inflammatory reactions in long-term experimental models.

## 5. Conclusions

C-Point represents an innovative material for root canal obturation which expands under moisture conditions to improve sealing while adapting well to the canal structure. SEM-EDS analysis revealed distinct elemental compositions: C-Point primarily contained zirconium and cobalt ions, while gutta-percha cones demonstrated a strong zinc signature with trace amounts of barium, aluminum, and sulfur. Histologically, no significant difference in inflammatory response was observed between C-Point and gutta-percha at any time point. The biocompatibility level of C-Point matches that of gutta-percha, but researchers must investigate its mechanical properties alongside durability and the ability to perform retreatments. Endodontic obturation materials research continues to advance through studies directed at developing materials that exhibit improved sealing properties while maintaining biological compatibility and enduring clinical performance. Clinical studies that assess C-Point’s effectiveness and tissue interaction with handling features need to be completed thoroughly to prove its validity as an alternative to traditional obturation products for modern endodontics. Finally, future research should be further evaluated, focused on the evaluation of mechanical resistance under clinical stress, the monitoring of long-term ion release, and the exploration of interactions with various endodontic sealers.

## Figures and Tables

**Table 1:** Inflammatory infiltrate scores (median and range) at 30- and 60-day evaluation periods. Lowercase superscript letters within each column denote statistically significant intergroup differences (*p* < 0.05, ANOVA/Tukey test).

Material	30 Days	60 Days
**Gutta-percha**	1.50 ^a^ (1.00–2.00)	1.50 ^a^ (1.00–2.00)
**C-Point**	2.00 ^a^ (1.00–2.00)	2.00 ^a^ (1.00–3.00)

## Data Availability

The raw data supporting the conclusions of this article will be made available by the authors on request.
